# ΔNp73, TAp73 and Δ133p53 Extracellular Vesicle Cargo as Early Diagnosis Markers in Colorectal Cancer

**DOI:** 10.3390/cancers13092240

**Published:** 2021-05-07

**Authors:** Javier Rodríguez-Cobos, David Viñal, Carmen Poves, María J. Fernández-Aceñero, Héctor Peinado, Daniel Pastor-Morate, Mª Isabel Prieto, Rodrigo Barderas, Nuria Rodríguez-Salas, Gemma Domínguez

**Affiliations:** 1 Department of Biochemistry, Faculty of Medicine, Health Research Institute Alberto Sols CSIC-UAM, IdiPaz, 28029 Madrid, Spain; jrcobos@iib.uam.es; 2 Department of Medical Oncology, Hospital Universitario La Paz, CIBERONC, 28046 Madrid, Spain; dvinal@alumni.unav.es (D.V.); nuria.rodriguez@salud.madrid.org (N.R.-S.); 3 Gastroenterology Unit, Hospital Clínico San Carlos, 28040 Madrid, Spain; carmenpoves@yahoo.es; 4 Surgical Pathology Department, Hospital Gregorio Marañón, 28007 Madrid, Spain; mgg10167@gmail.com; 5 Microenvironment and Metastasis Laboratory, Molecular Oncology Program, Spanish National Cancer Research Center, 28029 Madrid, Spain; hpeinado@cnio.es; 6 Surgery Department, Hospital Universitario La Paz, 28046 Madrid, Spain; danielpastormorate@gmail.com (D.P.-M.); iprieto@intermic.com (M.I.P.); 7 Chronic Disease Programme (UFIEC), Instituto de Salud Carlos III, Majadahonda, 28222 Madrid, Spain; r.barderasm@isciii.es

**Keywords:** ΔNp73, TAp73, Δ133p53, colorectal cancer, extracellular vesicles, liquid biopsy, screening programs, biomarkers, early diagnosis, premalignant lesions

## Abstract

**Simple Summary:**

The survival of colorectal cancer patients largely relies on the stage at diagnosis. The identification of early and non-invasive biomarkers to be used in screening programs for the diagnosis of the disease at the premalignant stage is mandatory. The aim of this study is to validate in plasma-derived extracellular vesicles secreted by malignant cells the diagnostic potential of well-known tumor-associated genes, ΔNp73, TAp73, and Δ133p53, in healthy subjects (*n* = 29), individuals with premalignant lesions (*n* = 49), and colorectal cancer patients (*n* = 42). Our data support ΔNp73 levels contained in extracellular vesicles as such a non-invasive and premature biomarker for the early diagnosis of colorectal cancer.

**Abstract:**

The early diagnosis of colorectal cancer is a key factor in the overall survival of the patients. The actual screening programs include different approaches with significant limitations such as unspecificity, high invasiveness, and detection at late stages of the disease. The specific content of extracellular vesicles derived from malignant cells may represent a non-invasive technique for the early detection of colorectal cancer. Here, we studied the mRNA levels of ΔNp73, TAp73, and Δ133p53 in plasma-derived extracellular vesicles from healthy subjects (*n* = 29), individuals with premalignant lesions (*n* = 49), and colorectal cancer patients (*n* = 42). Extracellular vesicles’ ΔNp73 levels were already significantly high in subjects with premalignant lesions. Δ133p53 levels were statistically increased in colorectal cancer patients compared to the other two groups and were associated with patients’ survival. Remarkably, TAp73 mRNA was not detected in any of the individuals. The evaluation of ΔNp73, Δ133p53 and CEA sensitivity, specificity and AUC values supports ΔNp73 as a better early diagnosis biomarker and CEA as the best to identify advanced stages. Thus, low levels of CEA and a high content of ΔNp73 may identify in screening programs those individuals at higher risk of presenting a premalignant lesion. In addition, Δ133p53 emerges as a potential prognosis biomarker in colorectal cancer.

## 1. Introduction

Colorectal cancer (CRC) is the third most common tumor diagnosed and accounts for more than 10% of all tumor types worldwide. It is estimated that CRC is the fourth leading cause of cancer-related death, with around 600,000 deaths reported annually [1,2].

CRC arises due to the accumulation of genetic alterations following a stepwise process from normal colonic epithelium to premalignant lesions (adenomatous and serrated polyps) and invasive cancer [3]. The identification and removal of premalignant lesions prevent the development of invasive CRC and constitute the basis for the screening programs. Fecal occult blood test (FOBT), fecal immunochemical testing (FIT), flexible sigmoidoscopy, and colonoscopy [4] are the tests included in screening programs and international guidelines [1,4,5,6]. However, although a benefit in incidence and survival has been reported, each technique has limitations. For colonoscopy, major disadvantages include the inconvenience of bowel preparation, the risk of perforation, bleeding, and infection, and those associated with sedation. For FOBT and FIT, the sensitivity for premalignant lesions is low (5–40%) [7,8] and unnecessary colonoscopies may be performed in case of false positive results. The identification of biomarkers through a non-invasive approach that could identify the disease at the premalignant stage is an appealing field of research for the early diagnosis of cancer. Available blood-based markers such as carcinoembryonic antigen (CEA) are not useful to detect early CRC due to significant overlap with benign disease, with a sensitivity that only reached 46% in a recent meta-analysis [9]. 

In recent years, liquid biopsy, referred to as the isolation of cancer-derived components from biological fluids, has been studied in several clinical scenarios, including cancer screening [10]. These components include, among others, circulating tumor cells, cell-free DNA, and extracellular vesicles (EVs). EVs are enriched in proteins, nucleic acids, and lipids that constitute a novel mode of intercellular communication and play an important role in cancer [11,12]. A recent meta-analysis found a good performance for CRC diagnosis of all liquid biopsy methods, with a sensitivity, specificity, and area under the curve (AUC) of 0.77, 0.89 and 0.90, respectively. The diagnostic performance of EVs in detecting CRC was 0.76, 0.92, and 0.9037. The role of liquid biopsy in predicting premalignant lesions remains understudied [13].

The expression of *TP73*, a member of the *TP53* family, is altered in most human cancers and has prognostic implications [14]. *TP73* translates into different variants with opposing functions: TAp73 and ΔTAp73 (ΔEx2p73, ΔEx2/3p73, ΔNp73 and ΔN’p73) [14]. In patients with CRC, the overexpression of ΔTAp73 isoforms has been associated with advanced tumor stage, shorter survival [15,16,17] and drug resistance [16]. Interestingly, our group found that the amount of ΔNp73 packaged in tumor-derived exosomes is also associated with survival [18]. Finally, the dysregulation of *TP73* has been reported in premalignant lesions of other tumor types [19,20,21,22]. Interestingly, the N-terminal truncated *TP53* variants have been also described and attracted during the last few years the interest of the scientific community due to their putative oncogenic properties and association with cancer progression [23,24,25].

Thus, similar to ΔNp73, the N-terminal truncated *TP53* variant Δ133p53 may function as an oncogene by acting as a dominant-negative inhibitor of other p53 family members and transactivating a specific set of pro-tumoral effectors [26,27,28]. Accordingly, Δ133p53 may transactivate DNA-repair genes [29,30] and favor resistance processes, regulate the JAK-STAT3 and RhoA-ROCK signaling pathways promoting cell growth and invasion [31] and modulate pro- and antiangiogenic factors [32]. In addition, Δ133p53 associates with an enhanced cancer stem cell phenotype and may induce the epithelial to mesenchymal transition (EMT) in cancer cells [33] and favor an immunosuppressive environment [34]. Δ133p53 has been observed to be upregulated in several tumor types, such as colorectal, lung, breast, prostate and esophageal squamous cell carcinomas and melanoma, and has been associated with poorer survival rates [28].

The deregulated expression of ΔNp73 and Δ133p53 in different tumor types and its association with cancer patients’ outcome support them as potentially good cancer early diagnostic candidates to further explore in plasma-derived EVs. The content of tumor suppressors in EVs as tumor biomarkers has been poorly explored; therefore, TAp73, which has previously been found overexpressed in different tumor types but controversially associated with patients’ outcome [14], could provide interesting information regarding tumor-derived EVs’ cargo and its clinical usefulness.

With this background, we evaluate here the mRNA levels of ΔNp73, TAp73 and Δ133p53 in plasma-derived EVs from healthy subjects (*n* = 29), individuals with premalignant lesions (PL) (*n* = 49), and CRC patients at different stages of the disease (*n* = 42) in order to explore their potential role in the early diagnosis of CRC through a non-invasive approach.

## 2. Materials and Methods

### 2.1. Samples and Subjects

The study was approved by the Research Ethics Board of the Hospital Clínico San Carlos and Hospital Universitario La Paz (Madrid, Spain). Blood samples were obtained with the corresponding informed written consent. Plasma samples were collected from 29 healthy individuals with negative colonoscopy, 49 subjects with premalignant lesions (low- and high-grade adenomas) and 42 CRC patients diagnosed at different stages of the disease. Sample size varied from 3–5 mL of plasma for our experimental workflow. 

Among these samples, 15/29 of control samples, 19/49 of adenoma samples, and 9/42 of CRC samples were additionally used to study their humoral response in a previous work [35]. The clinical data of the subjects included in the study are shown in Table 1. The plasma levels of CEA were available from 20 healthy subjects, 27 subjects with PL and 31 patients with CRC.

### 2.2. Extracellular Vesicles Isolation 

**Figure 1:**
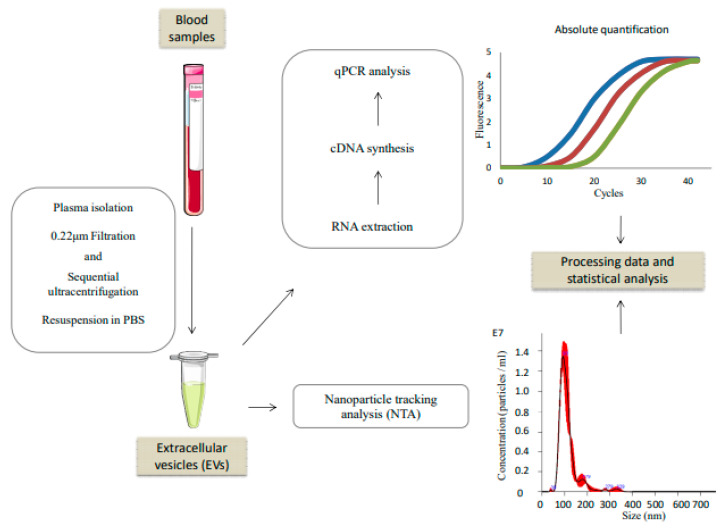
Scheme of the experimental workflow.

Plasma-derived EVs were isolated by a modified and adapted protocol of sequential ultracentrifugation previously described in [18]. Identification and characterization were performed as [18]. All centrifugations were performed at 10 °C with a Beckman 70.1 Ti rotor. The final EVs’ pellet was resuspended in PBS for subsequent procedures. A schematic representation of the workflow is in Figure 1.

### 2.3. Nanoparticle Tracking Analysis (NTA)

The NanoSight NS500 instrument equipped with a 405 nm laser (Malvern Panalytical Ltd, Malvern, UK) was calibrated using Silica Microspheres beads before measurements. Samples were diluted 1/1000 in PBS in order to obtain a particle concentration between 10^8^ and 10^9^ particles/mL. Three repeated measurements of 60 s were taken per sample and the mean value was used to determine particle number. The temperature of the laser unit was controlled at 25 °C. NTA software measured the size distribution (ranging from 10 to 1000 nm) and concentration (particles/mL) of nanoparticles.

### 2.4. RNA Extraction, Reverse Transcription and qPCR

RNA from resuspended EVs was extracted by SeraMir^TM^ Exosome RNA Amplification kit (System Biosciences SBI, Mountain View, CA, USA) following the manufacturer’s instructions. cDNA synthesis was performed with the Transcriptor First-Strand cDNA Synthesis Kit (Roche Diagnostics SL, Barcelona, Spain). Quantitative real-time PCR was performed in a LightCycler 2.0 instrument using Fast Start DNA MasterPlus SYBR Green I kit (Roche) as previously described in [17]. Primer sets used for ΔNp73, TAp73 and Δ133p53 were the following: forward, 5′-TCGGTGACCCCGCACGGCAC-3′; reverse, 5′-GCGAGTGGGTGGGCACGCTG-3′ for ΔNp73; forward, 5′-GCACCACGTTTGAGCACCTCT-3′; reverse, 5′-GCAGATTGAACTGGGCCATGA-3′ for TAp73; and forward, 5′-TGTCTCCTTCCTCTTCCTACAG-3′; reverse, 5′-ACCATCGCTATCTGAGCAGC-3′ for Δ133p53.

### 2.5. Statistical Analysis

To normalize the results, qPCR data were related to the number of particles of each sample. Then, these data were modified with log10 transformation. The D’Agostino–Pearson and Shapiro–Wilk tests were used to assess the normality of the data distribution. Subsequently, the Kruskal–Wallis and U-Mann–Whitney tests were used for statistical analysis. The receiver operating characteristic (ROC) curves were constructed with the R program (version 4.0.3, https://www.r-project.org/; accessed on: 1 October 2020), and the corresponding area under the curve (AUC) and the maximized sensitivity and specificity values were calculated using the R packages ModelGood and Epi. The cut-off value for the survival analysis was selected upon ROC curves for disease recurrence or death in patients with localized CRC and death for the whole cohort of patients with CRC. The optimal cut-off value was determined using the Youden’s J index. For ΔNp73, we selected 0.76 and −0.83 as the cut-off values for disease-free survival (DFS) and overall survival (OS), respectively. For Δ133p53, we selected 2.93 and 1.99 as the cut-off values for DFS and OS, respectively. ΔNp73 and Δ133p53 mRNA levels were classified in low and high levels accordingly. We used the log-rank test to compare DFS and OS according to ΔNp73 and Δ133p53 mRNA levels. Kaplan–Meier analyses were used to estimate medians. A Cox proportional-hazards model was used to estimate hazard ratios and 95% confidence intervals. The Spearman rank-order correlation test was used to examine the relationship between autoantibodies and EVs mRNA of the different p53 family isoforms. *p*-values ≤ 0.05 were considered statistically significant. Statistical analysis was performed with SPSS software (version 24).

## 3. Results

### 3.1. TAp73, ΔNp73, and Δ133p53 EVs Content in Control, PL and CRC Individuals

In order to evaluate the early diagnosis potential of the TAp73, ΔNp73, and Δ133p53 isoforms in CRC, we have quantified their mRNA levels on isolated EVs from healthy individuals, subjects with PL, and CRC patients diagnosed at different stages. NTA analysis did not show significant differences in EVs’ size distributions or the concentration of particles among the three groups and the samples of all groups exhibited similar EV size profiles (Figure S1).

**Figure 2:**
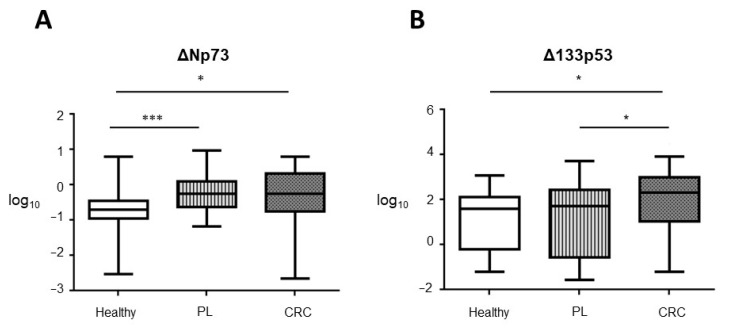
EVs’ mRNA levels of (**A**) ΔNp73 and (**B**) Δ133p53 in the three groups of individuals. Box and whisker plot displaying the qPCR data related to the EVs’ concentration. Statistical significance * *p* < 0.05; *** *p* < 0.001.

TAp73 mRNA levels were undetectable in any of the studied groups. Accordingly, we have considered the EV content of TAp73 mRNA as negative for all the samples. mRNA levels for ΔNp73 and Δ133p53 were detected in the three conditions. Specifically, ΔNp73 levels were significantly higher in subjects with PL and CRC patients than healthy controls (Figure 2A), although there were no significant differences between subjects with PL and CRC patients. Δ133p53 levels were significantly increased in EVs derived from CRC patients compared to the other two groups (Figure 2B).

### 3.2. Potential Value of ΔNp73 and Δ133p53 Versus CEA as Diagnostic Biomarkers

**Figure 3:**
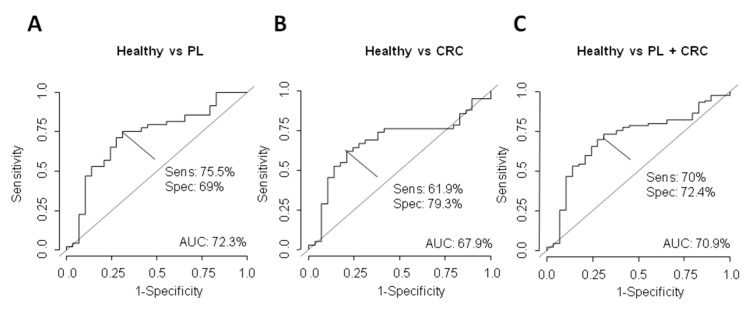
Determination of the ΔNp73 EV mRNA content as discriminating plasma biomarker between healthy and PL group (**A**), healthy and CRC patients (**B**) and healthy versus PL+CRC subjects (**C**) was carried out through ROC curves calculating AUC, its specificity (spec) and sensitivity (sens).

**Figure 4:**
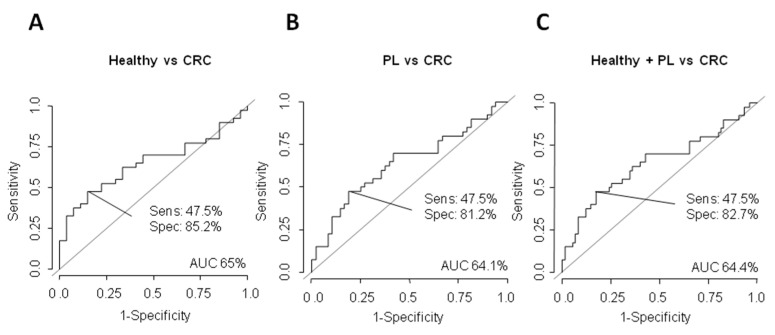
Determination of the Δ133p53 EV mRNA content as discriminating plasma biomarker between healthy and CRC patients (**A**), PL individuals and CRC patients (**B**) and healthy + PL subjects and CRC patients (**C**) was carried out through ROC curves calculating AUC, its specificity (spec) and sensitivity (sens).

In addition, we determined the usefulness as candidate biomarkers in plasma of ΔNp73 and Δ133p53, calculating their individual AUC, sensitivity and specificity by ROC curves (Figure 3 and Figure 4). Individual AUC values for ΔNp73 for discriminating PL from healthy subjects were 72.3% (sensitivity 75.5% and specificity 69%) (Figure 3A), 67.9% (sensitivity 61.9% and specificity 79.3%) for CRC patients from healthy subjects (Figure 3B) and 70.9% (sensitivity 70% and specificity 72.4%) for healthy subjects from subjects with PL and CRC patients (Figure 3C).

In the case of Δ133p53, individual AUC values for discriminating CRC patients from healthy subjects were 65% (sensitivity 47.5% and specificity 85.2%) (Figure 4A), 64.1% (sensitivity 47.5% and specificity 81.2%) for CRC patients from subjects with PL (Figure 4B) and 64.4% (sensitivity 47.5% and specificity 82.7%) for healthy and PL subjects from CRC patients (Figure 4C).

**Figure 5:**
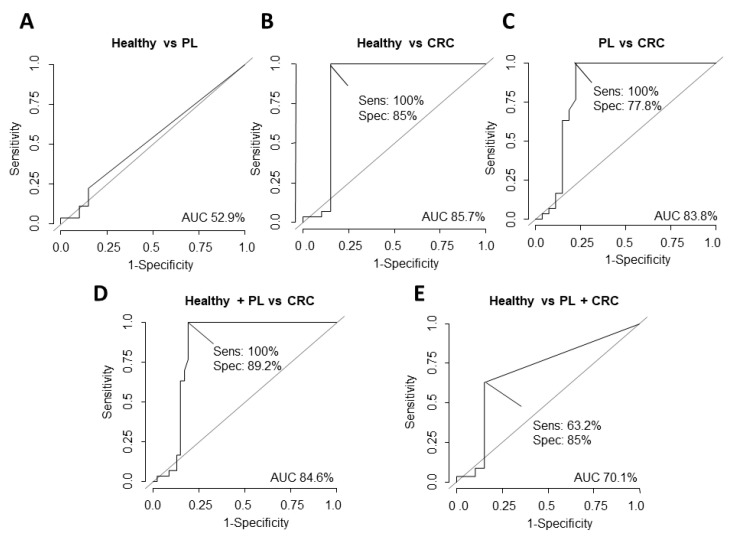
Determination of the diagnostic potential of CEA levels in plasma to discriminate healthy subjects versus subjects with PL (**A**), healthy subjects versus patients with CRC (**B**), subjects with PL versus patients with CRC (**C**), healthy + PL subjects versus patients with CRC (**D**), and healthy subjects versus PL subjects + patients with CRC (**E**) carried out through ROC curves calculating AUC, its specificity (spec) and sensitivity (sens).

Finally, we decided to compare the potential of ΔNp73 and Δ133p53 versus CEA to discriminate among the three groups. CEA AUC values for discriminating healthy subjects from PL subjects were 52.9% and 85.7% (sensitivity 100% and specificity 85%) from healthy subjects and CRC (Figure 5). As previously described in the literature, our data do not support CEA as a good marker to discriminate between healthy and PL subjects [9]. In this sense and based on the AUC values, ΔNp73 emerges as a much better marker than CEA to discriminate between these two groups (Figure 3A and Figure 5A). However, neither ΔNp73 nor Δ133p53 improve the ability of CEA to discriminate between healthy subjects and CRC patients (Figure 3B, Figure 4A and Figure 5B).

### 3.3. Correlation with Tumoral Stage, DFS and OS

**Figure 6:**
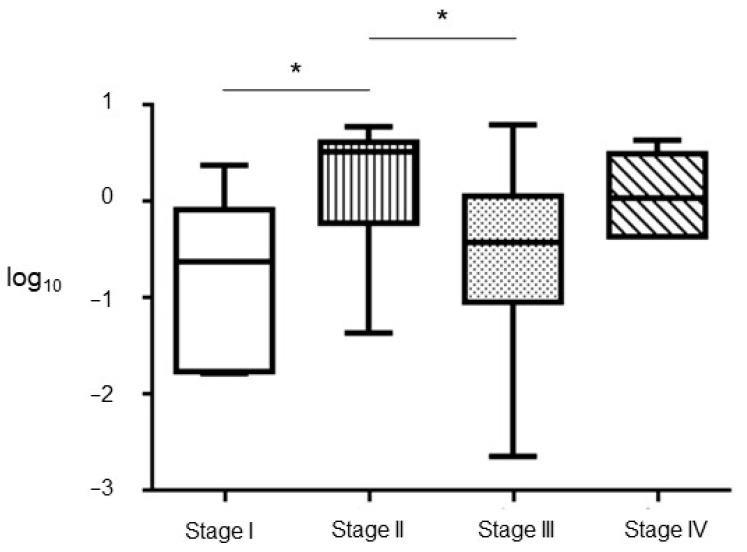
ΔNp73 mRNA contained in the EVs according to tumoral stage. Box and whisker plot displaying the qPCR data related to the EVs’ concentration. Statistical significance, * *p* < 0.05.

Next, we have evaluated the EVs’ mRNA content of these isoforms at the different stages of the disease. Non statistical significative differences were observed for Δ133p53. However, ΔNp73 was observed to be increased at stage II and IV, although it did not reach statistical significance at this later stage (Figure 6).

In the CRC cohort, the median follow-up was 36.2 months (range, 1.1 to 62.4). During this period, 15 (34.9%) events (recurrences or deaths) were observed among patients with localized disease (*n* = 38) and 16 (37.2%) deaths were observed in the whole cohort (*n* = 42). At 3 years, 63% (95% confidence interval (CI), 47 to 78) of the patients with localized disease remained alive without disease recurrence and 69% (95% CI, 55 to 83) remained alive in the whole cohort.

**Figure 7:**
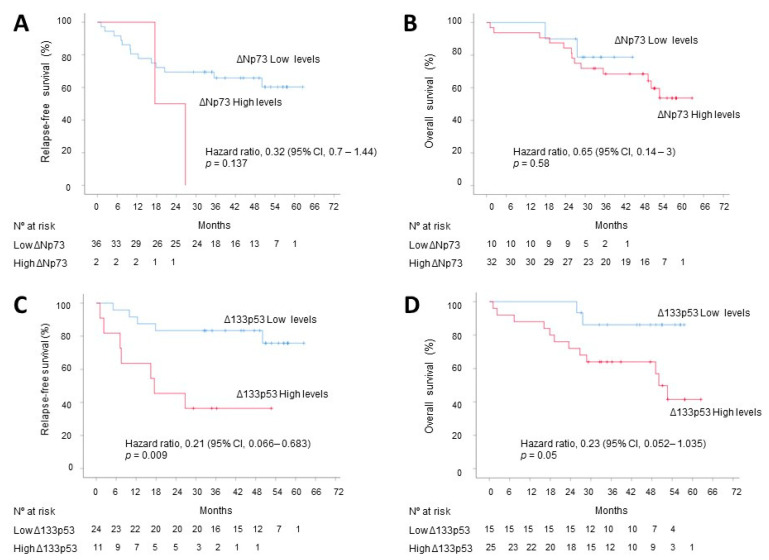
Kaplan–Meier estimates of DFS and OS. Influence of EVs’ content of ΔNp73 on DFS (**A**) and OS (**B**), and EVs’ content of Δ133p53 on DFS (**C**) and (**D**) on OS. Tick marks indicate censored data. Low and high levels of ΔNp73 and Δ133p53 were classified according to the selected cut-offs.

Survival analysis did not reveal a correlation between ΔNp73 EV content and DFS and OS (Figure 7A,B). However, Δ133p53 EV content was inversely associated with DFS and OS (Figure 7C,D). The median DFS was not reached in the low Δ133p53 group and was 17.5 months (95% CI, 0 to 38) in the high Δ133p53 group, with a hazard ratio (HR) for disease recurrence or death of 0.21 (95% CI, 0.06 to 0.68; *p* = 0.009) in favor of the high Δ133p53 group. In the whole cohort of CRC, patients with low Δ133p53 levels did not reach the median OS and patients with high Δ133p53 levels had a median OS of 50 months (95% CI, 44.9 to 55.2), with a HR of 0.23 (95% CI, 0.05 to 1.03; *p* = 0.05).

### 3.4. Correlation between Seroreactivity of p53 Family Members and EVs Content

We previously analyzed the potential seroreactivity of different variants of the p53 family (p53, p73, ΔNp73α, and ΔNp73β) [35] in a subset of our current cohort (healthy, *n* = 15; PL, *n* = 19; CRC, *n* = 9). Here, the correlation between the autoantibody levels and the ΔNp73 and Δ133p53 EV content was evaluated. We have observed a negative correlation trend in ΔNp73α seroreactivity and ΔNp73 EV levels in patients with CRC (r = −0.548; *p* = 0.09) (Table 2).

## 4. Discussion

CRC screening is currently performed by different techniques which present significant limitations. On one hand, the fecal occult blood test shows a lack of specificity and a low sensitivity for early stages and premalignant lesions. On the other hand, colonoscopy, although with high specificity and sensitivity, is highly invasive and adherence of the general population is relatively low. Therefore, the detection of soluble biomarkers from different body fluids, known as liquid biopsy, as a non-invasive technique emerges as a promising approach for the early diagnosis of the disease. In this sense, the analysis of the cargo of plasma EVs released by the tumor and by premalignant lesions in apparently healthy subjects is of great interest and remains largely unexplored.

The overexpression of different *TP53* family members in human cancers and its association with a poor prognosis of the patients have been described. Thus, ΔNp73 upregulation is associated with shorter survival in hepatocellular carcinoma [36], neuroblastoma [37], lung cancer [38], gastric and esophageal carcinoma [39], acute promyelocytic leukemia [40], and colorectal adenocarcinoma [16]. ΔNp73 has also been detected in EVs from CRC patients and associated with a worse outcome [18]. Interestingly, although TAp73 presents tumor suppressor abilities similar to those described for *TP53*, it has been described as overexpressed in many human tumors including CRC and associated with poorer survival [15,41,42,43,44]. Remarkably, its status in EVs from cancer patients has not been tested yet. The N-terminal truncated *TP53* variant, Δ133p53, has attracted during the last few years the interest of the scientific community due to its putative oncogenic properties. Δ133p53 may act as a survival factor [23], inhibits senescence [30], interferes with p53-mediated apoptosis [25,29], induces chemoresistance [24], and promotes invasion capacity through the epithelial–mesenchymal transition [33]. Similar to ΔNp73, Δ133p53 may function as an oncogene by acting as a dominant-negative inhibitor of other p53 family members and transactivating a specific set of pro-tumoral effectors [26,28], such as DNA-repair genes [29,30], the JAK-STAT3 and RhoA-ROCK signaling pathways [31], pro- and antiangiogenic factors [32] and EMT and stem genes. Δ133p53 has been observed to be upregulated in several tumor types, such as colorectal, lung, breast, prostate and esophageal squamous cell carcinomas and melanoma, and associated with poorer survival rates [28]. Additionally, Arsic N et al. associated Δ133p53 upregulation with a higher risk of metastatic recurrence in CRC patients [25]. Remarkably, Δ133p53 expression has been observed to be reduced in adenomas compared to control samples but increased in adenocarcinoma tissues [30]. 

In the present study, we report statistically significant high levels of ΔNp73 in plasma EVs in both subjects with CRC and with PL compared with healthy individuals. The comparison of AUC values, specificity and sensitivity addressed by ROC curves between ΔNp73 in EVs and CEA levels in plasma in the three groups of study clearly revealed a much greater potential of ΔNp73 to identify individuals with PL. Our findings highlight the plausible usefulness of ΔNp73 to the detriment of CEA as a non-invasive biomarker for the detection of the disease at the premalignant stage, probably playing a key role in the initiation of the tumor. It is noteworthy that EVs’ cargo can be uptaken by different cells of the cancer microenvironment, leading to a pro-tumoral stroma [18,45,46]. In this sense, we previously described that ΔNp73 contained in EVs can be selectively taken up by fibroblasts and endothelial cells [18], which may support its putative role modulating the microenvironment. Further studies supporting this statement are needed.

We expected low levels of TAp73 in the EVs from PL individuals and CRC patients compared to healthy subjects. Surprisingly, TAp73 was not detected in any of the groups evaluated in the current study. These findings suggest that packaging and secreting tumor suppressor mRNAs in EVs may not provide a selective advantage to the tumor. The latter has to be confirmed since this is the first study evaluating the EV content of TAp73. In addition, many studies analyze the pro-tumoral cargo of EVs and its function in tumor progression and conditioning of the premetastatic niche, but few evaluate its diagnostic and prognosis biomarker potential [45,47].

To the best of our knowledge, this is also the first report evaluating the presence of Δ133p53 in EVs in CRC. Here, we observed higher levels of Δ133p53 in plasma-derived EVs from CRC patients than those from healthy subjects and individuals with PL. Fujita K et al. have previously reported the status of Δ133p53 in tissues from healthy, PL and CRC subjects [30]. Although their results in CRC patient tissues are similar to those observed in our study in EVs, we did not observe its down-expression in EVs from PL individuals compared to controls. The differences observed in both studies are probably due to the fact that EV content may not recapitulate the tumor status and EVs’ cargo is actively packaged. The over-representation of Δ133p53 in EVs from CRC supports its role in the progression of the disease and its usage as a tumor biomarker, but in contrast to ΔNp73 that is already overexpressed in plasma-derived EVs from PL subjects, Δ133p53 may not be of clinical use for the early detection of the disease since its levels in individuals with PL are similar to those of healthy subjects. Remarkably, the AUC values, specificity and sensitivity analyzed for ΔNp73, Δ133p53 and CEA support the levels of CEA in plasma as a better marker of patients with advanced CRC than ΔNp73 and Δ133p53 content in EVs. These data point to a combination of CEA and ΔNp73 to discriminate among healthy individuals, subjects with PL and CRC patients in the context of a screening of the general population. Those individuals with low CEA plasma levels and high ΔNp73 in EVs are more probable to present PL. However, we must be cautious with the usage of CEA in the clinical setting. CEA is a widely soluble biomarker that has validated its usefulness to detect disease recurrence in postoperative surveillance [48]. Its potential as a screening biomarker is dismissed due to its low sensitivity in early stages of CRC in concordance with the data we present here when comparing CEA and ΔNp73 sensitivity, specificity and AUC values. Different studies also reported a low sensitivity for CEA in patients at early stages of CRC [49,50,51]. It is noteworthy that CEA is associated with other types of cancer and malignancies [52], and the sensitivity and specificity depend on the used immunoassays and the tested population [53,54]. Consequently, the performance of CEA in the early diagnosis of the disease is poor, which supports the need to identify new biomarkers for the early detection of the malignancy, as is the case of ΔNp73. Nevertheless, our results suggest a better potential of CEA versus ΔNp73 and Δ133p53 in the identification of advanced CRC.

We have observed that those CRC patients at stage II and IV of the disease present higher levels of ΔNp73. These findings are similar to those previously reported [18] regarding the upregulation of ΔNp73 in EVs at advanced stages of CRC and its association with a shorter survival. This observation could be related to the fact that ΔNp73 has been associated with the progression of the disease and with resistance to treatments, conferring a clear advantage to the tumor [14]. The fact that we observed here an upregulation of ΔNp73 at stage II is intriguing, although it could be assumed that the release of this isoform at this stage may contribute to the local dissemination of the tumor. Stage II CRC patients do not benefit from the current chemotherapeutic regimens; however, there is a significant percentage of these individuals that relapse. It could be interesting to evaluate whether those patients at stage II who present high ΔNp73 EV content are more prone to progress. This finding may support the usefulness of ΔNp73 EV levels as a marker of stage II patients susceptible to receive treatment.

No correlation was observed between ΔNp73 EV content and DFS and OS in our series of CRC patients. This is in contrast with previous results in which we observed a trend with survival [18]. The smaller size of the current series could explain these differences. Interestingly, high Δ133p53 content in EVs in the cancer group is associated with both DFS and OS. To our knowledge, this is the first report evaluating the implication of this p53 isoform contained in EVs in the prognosis of cancer patients. Our data support Δ133p53 as a potential candidate to explore further as a biomarker of tumor progression and patients’ release.

In our attempt to identify biomarkers for the early detection of CRC and the plausible usage of the *TP53* family members to this aim, we have recently described a significant difference in the seroreactivity for ΔNp73 between CRC patients and subjects with PL versus healthy controls [35]. Interestingly, we have not observed a correlation between ΔNp73 seroreactivity and EVs’ mRNA levels of ΔNp73, TAp73 and Δ133p53, although a trend is observed for the negative association between the levels of ΔNp73 autoantibodies and EVs’ ΔNp73 mRNA in patients with CRC. This finding may indicate that tumoral cells at advanced stages of the disease reduce their immunoresponse but enhance the release of EVs with specific cargo to communicate with the microenvironment, promoting its progression and probably conditioning the metastasis niche [45,55]. This is an appealing hypothesis that has to be confirmed in a larger cohort of patients.

## 5. Conclusions

This is the first report that studies the implication of ΔNp73, TAp73 and Δ133p53 content in plasma-derived EVs as early diagnosis biomarkers of CRCs, using for this purpose healthy subjects, individuals harboring premalignant lesions and CRC patients at different stages of the disease. Our data support the potential role of EVs’ ΔNp73 content to detect premalignant lesions as the earliest stage of the disease. Interestingly, although Δ133p53 cannot detect the disease at the premalignant stage, this isoform in EVs emerges as a potential prognosis marker in CRC. Further investigation could establish a combination with other non-invasive biomarkers and improve the actual screening programs of the disease, for instance, the combined levels of CEA in plasma and ΔNp73 in EVs as we describe here.

## Figures and Tables

**Table 1:** Clinical parameters of the subjects included in the study.

Parameters	Healthy Individuals	Subjects with PL	CRC Patients
*n*	29	49	42
Sex			
Male	10 (34.48%)	24 (48.98%)	22 (52.38%)
Female	19 (65.52%)	25 (51.02%)	20 (47.62%)
Age (years)			
Median, range	59 (42–79)	62 (31–81)	71 (49–89)
CEA (ng/mL)			
Median, range	0.0 (0.0–81.9)	0.0 (0.0–135.2)	3.1 (0.5–94.9)
Tumor stage			
I			5 (11.905%)
II			9 (21.43%)
III			23 (54.76%)
IV			5 (11.905%)
Tumor location			
Right colon			14 (33.33%)
Left colon			17 (40.48%)
Rectum			11 (26.19%)
DFS (*n* = 38)			
Median, CI			NR
3 years DFS (95%, CI)			75% (61–89)
OS			
Median, CI			NR
3 years OS (95%, CI)			69% (55–83)

*n*, number of subjects; DFS, Disease-free survival; OS, Overall survival; NR, Not Reached.

**Table 2:** Correlations between the seroreactivity of different variants of the p53 family (p53, p73, ΔNp73α, and ΔNp73β) and ΔNp73 and Δ133p53 EV content. The Spearman correlation coefficient (r) is shown in the table.

Seroreactiviy vs. EVs Content	Healthy Subjects	PL	CRC
p53 autoantibodies vs. EVs Δ133p53	0.194	−0.303	−0.339
p73 autoantibodies vs. EVs ΔNp73	−0.110	0.127	0.018
ΔNp73α autoantibodies vs. EVs ΔNp73	−0.064	0.050	−0.548
ΔNp73β autoantibodies vs. EVs ΔNp73	−0.064	0.018	−0.305

PL, premalignant lesions; CRC, colorectal cancer.

## Data Availability

The data presented in this study are available on request from the corresponding author. The data are not publicly available due to us continuing to enlarge the cohort.

## Supplementary Materials

The following are available online at https://www.mdpi.com/article/10.3390/cancers13092240/s1. Figure S1: Nanoparticle tracking analysis of plama-derived EVs.
