# Supplementary material for: ΔNp73, TAp73 and Δ133p53 Extracellular Vesicle Cargo as Early Diagnosis Markers in Colorectal Cancer
Source: Cancers (Basel). 2021 May 7;13(9):2240. doi: 10.3390/cancers13092240 (PMC8124369; doi:10.3390/cancers13092240)
Supplement: Supplementary file 1 [file cancers-13-02240-s001.zip › cancers-1182156-supplementary.pdf]

Supplementary Materials: ΔNp73, TAp73 and Δ133p53 Extracellular Vesicle Cargo as Early Diagnosis Markers in Colorectal Cancer

Javier Rodríguez-Cobos, David Viñal, Carmen Poves, María J. Fernández-Aceñero, Héctor Peinado, Daniel Pastor-Morate, Mª Isabel Prieto, Rodrigo Barderas, Nuria Rodríguez-Salas and Gemma Domínguez

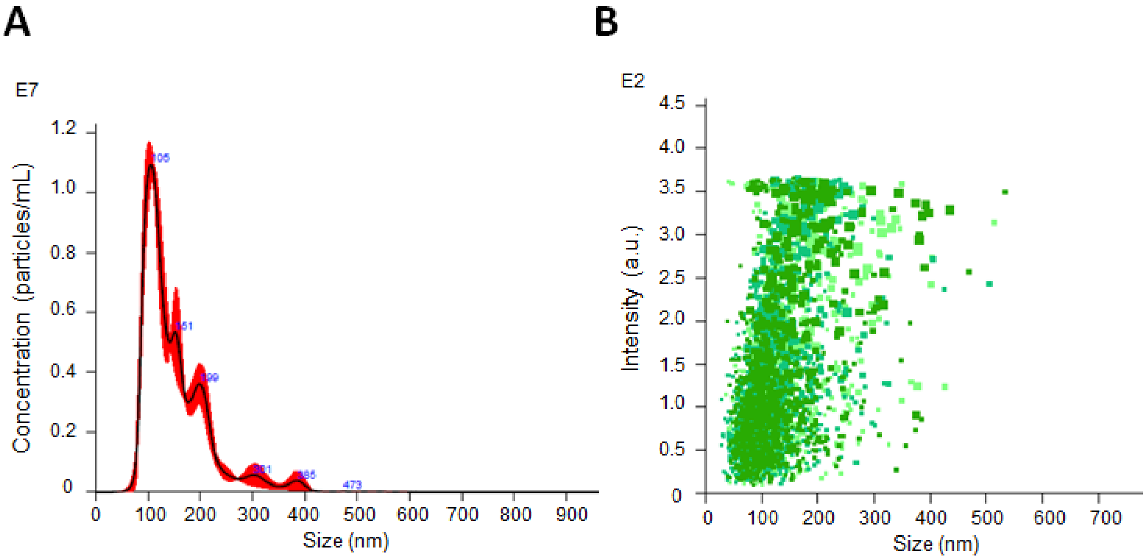

Figure 1. Nanoparticle tracking analysis of plasma-derived EVs. (**A**) Representative profile of particle concentration and size distribution. The red bar indicate the standard error of the mean value from the measurements (n = 3). (**B**) Representative profile of intensity (arbitrary units; a.u.) and size distribution. .

© 2020 by the authors. Submitted for possible open access publication under the terms and conditions of the Creative Commons Attribution (CC BY) license (http://creativecommons.org/licenses/by/4.0/).
